# Nrf2: new insight in cell apoptosis

**DOI:** 10.1038/cddis.2015.256

**Published:** 2015-10-08

**Authors:** M Bonay, T B Deramaudt

**Affiliations:** 1 INSERM U1179, Laboratoire de Physiologie TITAN, UFR des Sciences de la Santé—Simone Veil, Université de Versailles Saint-Quentin-en-Yvelines, Montigny-le-Bretonneux, France; 2 Service de Physiologie-Explorations Fonctionnelles, Hôpital Ambroise Paré, Assistance Publique-Hôpitaux de Paris, Boulogne, France; 3 LIA-BAHN (Laboratoire International Associé – Biologie Appliquée Handicap Neuromusculaire), CSM (Centre Scientifique de Monaco), Monaco

In a recent issue of *Cell Death and Discovery*, we described a new mechanism by which sulforaphane decreased mycobacterial burden in an *in vitro* model of THP-1-derived macrophage infection by *Mycobacterium abscessus*.^1^ This sulforaphane-mediated diminution was due to the induction of a caspase-independent cell apoptosis and necessitated activation of both nuclear factor E2-related factor 2 (Nrf2) and p38 mitogen-activated protein kinases (MAPK) signaling pathways.

*M. abscessus* is a nontuberculous mycobacterium that belongs to the group of rapid growing mycobacteria. This emerging pathogen is able to cause skin, bone and soft tissue infections, and more generally exacerbations of lung diseases.^2^ Its high tolerance to antibiotics, which limits patient treatment, has become a public health concern worldwide. *M. abscessus* has been increasingly involved in patients with cystic fibrosis and in immunosuppressed patients,^3^ enhancing their risk of developing chronic airway infections and leading to a fatal outcome.

Bacterial infection induces an imbalance between oxidants and antioxidants, triggering an oxidative burst that is well known for its bactericidal effect. Yet, some selected mycobacteria are able to thrive in this oxidative environment. It is then unsurprising that the use of oxidant scavengers such as MnTE-2-PyP or *N*-acetyl-L-cysteine has been shown to decrease *M. abscessus* load by activating bacterial killing in phagolysosomes.^4^ In our study, we decreased the oxidative environment in THP-1-derived macrophages by activating Nrf2, the key transcription factor that controls the cascade of cytoprotective and antioxidant defense mechanisms, and the maintenance of the redox homeostasis. Upon oxidative stress or infection, cytoplasmic Nrf2 is released from its inhibitor Keap-1, translocates into the nucleus, and forms a cofactor complex that binds to specific antioxidant responsive elements (ARE) found in promoters of phase II antioxidant and detoxifying enzymes. Nrf2 signaling pathway has been shown to have an important role whether beneficial or detrimental, in microbial infections.^5^

*M. abscessus* infection of THP-1-derived macrophages activates Nrf2 signaling pathway, and induces the expression of heme oxygenase-1 and NADPH quinone oxidoreductase-1, two downstream targets of Nrf2. In addition, *M. abscessus* induced reactive oxygen species (ROS) production in infected macrophages and blocked phagosomal acidification thus allowing its intracellular proliferation.

Sulforaphane, a well-established activator of Nrf2 was used to pretreat macrophages before mycobacterial infection. The results showed an inhibition of mycobacterial growth 7 days postinfection in sulforaphane pretreated macrophages compared with vehicle pretreated ones. This sulforaphane-induced mycobacterial growth inhibition was neither caused by phagosomal pH acidification nor increase in cell phagocytosis, but rather induction of cell apoptosis. Contrary to the well-known cyprotective effect of sulforaphane and Nrf2 signaling pathway,^6^ we demonstrated that pre-stimulation of Nrf2 signaling pathway by sulforaphane before *M. abscessus* infection triggered a caspase-independent cell apoptosis. Pretreatment of macrophages with sulforaphane alone had no significant effect on cell death. To our knowledge, this is the first study showing sulforaphane-induced Nrf2 triggers a caspase3/7-independent and p38 MAPK-dependent cell apoptosis in mycobacteria infected macrophages (Figure 1).

Cell death in macrophages has a critical role in the host response to mycobacterial infection. Cell apoptosis is an effective mechanism used by the innate defense system to fight bacterial infection. This energy-dependent mechanism generates apoptotic bodies that will facilitate T-dependent antigen response and induce mycobacterial killing by uninfected neighboring macrophages.^7^ As *M. abscessus* has only been recently identified as a separate species, very little is known in the dynamics between *M. abscessus* and macrophage cell survival/cell death. In our *in vitro* model, *M. abscessus* induced cell necrosis in untreated cells but not cell apoptosis. This phenomenon has been observed in *M. tuberculosis* that has the ability to inhibit cell apoptosis in favor of cell necrosis.^8^

Recent highlights on caspase-independent forms of apoptosis triggered by infection have been described in the literature. Necroptosis, also known as programmed necrosis, can be initiated by various agents including microbial infection, and is characterized by activation of the receptor-interacting protein (RIP) kinases RIP1 and RIP3-dependent signaling pathway. This programmed cell death is a critical antiviral process in the host innate immune defense system. Phagocytes infected with viruses that are able to inhibit the classical caspase-dependent apoptosis pathway can undergo necroptosis to facilitate viral clearance by the neighboring immune system.^9, 10^ Moreover, Roca and Ramakrishnan have demonstrated a tumor-necrosis factor (TNF)-induced necroptosis in a *M. tuberculosis* infected zebrafish model. In the early stages of mycobacteria infection, injection of an excess of TNF in infected macrophages induced mitochondrial ROS production through a RIP1/RIP3-dependent kinase pathway, process necessary for the bacterial clearance. However, ROS-mediated necroptosis rapidly led to release and propagation of *M. tuberculosis* in the extracellular microenvironment.^11^

Another caspase 3/7-independent apoptosis is the inflammation-mediated cell death known as pyroptosis. This particular programmed cell death participates in the host cell defense system against pathogens and requires activation of caspase 1, IL-1*β* and IL-18.^12^ Recently, Bai *et al.*^13^ have shown that *M. tuberculosis*-mediated production of IL-32*γ* in THP-1-derived macrophages induced caspase 1-dependent pyroptosis which help reduce intracellular *M. tuberculosis* burden.

In conclusion, additional studies are required to specifically identify the caspase-independent form(s) of apoptosis initiated by the combination of Nrf2 activation and *M. abscessus* infection. Nevertheless, our findings strongly suggest that Nrf2 activators may be of great interest as future therapeutic treatments in supplement to the actual lengthy multi-drug therapies used in patients diagnosed with mycobacterial infection.

## Figures and Tables

**Figure 1:**
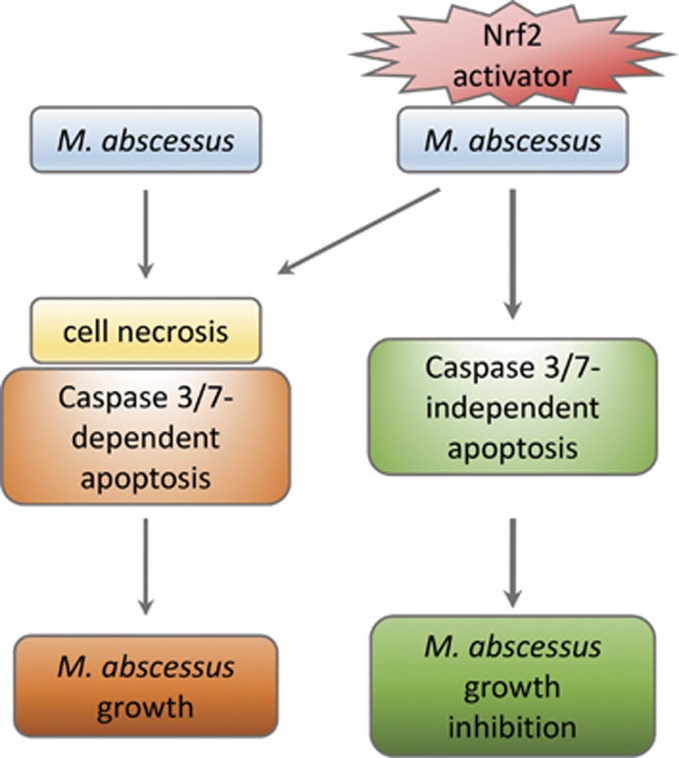
Infection of THP-1 derived macrophages by *M. abscessus* elicits caspase3/7-dependent cell apoptosis. Pretreatment of macrophages with an Nrf2 activator before mycobacterial challenge initiates a caspase-independent cell apoptosis

## Abbreviations

MAPK: mitogen-activated protein kinases
Nrf2: Nuclear factor E2-related factor 2
ROS: reactive oxygen species
TNF: tumor-necrosis factor
